# Prevalence and impact of sleep disorders in osteoarthritis: A systematic review and meta-analysis

**DOI:** 10.1016/j.ocarto.2025.100738

**Published:** 2025-12-24

**Authors:** Sylvain Mathieu, Anne-Christine Rat, Jérémie Sellam

**Affiliations:** a University of Clermont Auvergne, CHU Clermont-Ferrand, Inserm, Neuro-Dol, Clermont-Ferrand, France; b Department of Rheumatology, CHU Clermont-Ferrand, Clermont-Ferrand, France; c Normandie Univ, UNICAEN, CHU de Caen Normandie, Rheumatology Department, Inserm, COMETE, 14000 Caen, France; d Department of Rheumatology, Assistance Publique - Hôpitaux de Paris (AP-HP), Saint-Antoine Hospital, France; e Sorbonne Université, INSERM UMRS 938, Centre de Recherche Saint-Antoine (CRSA), Paris, France

**Keywords:** Sleep disorders, Osteoarthritis, Meta-analysis, Systematic literature review, Pain

## Abstract

**Objective:**

Sleep disorders (SD) are increasingly recognized as a major contributor to the symptom burden of osteoarthritis (OA), yet their epidemiology and clinical impact remain insufficiently characterized. This systematic literature review with meta-analysis aims to quantify the prevalence of SD in OA patients and to assess their influence on patient-reported outcomes.

**Method:**

We searched the PubMed, Embase, Web of Science and Cochrane Library databases up to 22 July 2025 (PROSPERO CRD120251067310). The Pittsburgh Sleep Quality Index (PSQI), sleep efficiency, and the number of patients with SD, including insomnia, sleep apnea and restless legs syndrome (RLS) were collected. The prevalence of SD was calculated using metaproportion. Differences were measured using the inverse variance method or the Mantel–Haenszel method.

**Results:**

We selected 81 articles representing 289,914 patients, including 75,129 knee, 708 hand and 236 hip OA. For the remaining patients, OA location was not noticed or multiple. The prevalence of SD was 68.9 % (63 studies, 276,092 patients), 32.0 % for sleep apnea, 34.0 % for insomnia and 51.6 % for RLS. Compared to healthy controls, the prevalence of SD was higher in OA patients which had poorer sleep quality with a higher PSQI score (7.9 ± 3.4 vs. 6.5 ± 2.7; p = 0.002) and lower sleep efficiency (82.1 ± 12.5 vs. 89.8 ± 6.0 %; p = 0.02). OA patients with SD were younger, had a higher BMI, a greater pain intensity and a higher frequency of depression and catastrophizing.

**Conclusion:**

Sleep disorders are common in OA patients and should be investigated and managed since they have a negative impact on pain, fatigue and disability.

**Keypoints**

1) Sleep disorders are frequent in osteoarthritis
2) Sleep disorders in OA are associated with greater pain, fatigue, and psychological distress.
3) Systematic assessment and management of sleep disorders should be part of OA care.

## 1 Introduction

Osteoarthritis (OA) is the most common form of arthritis and a leading cause of chronic pain and functional disability worldwide [1,2]. In addition to joint-related symptoms, patients living with OA often experience other symptoms such as fatigue, anxiety-depression, pain catastrophizing and/or sleep disturbances. Sleep disorders can be particularly troublesome for patients with chronic pain, especially those with painful rheumatic diseases [3,4]. According to international classification [5], sleep disorders include insomnia, obstructive sleep apnea, restless legs syndrome and other sleep problems, such as not feeling rested or experiencing daytime sleepiness.

In patients with OA, sleep disorders can exacerbate pain, fatigue, and mood, as well as impairing physical function and quality of life [6]. In a recent meta-analysis of qualitative studies, the GO-PAIN working group found that OA patients frequently experience sleep disorders with very illustrative verbatim such as “sharp pain comes on at night and makes it difficult to sleep”. Several patients reported being discouraged and greatly impacted by OA pain and sleep disorders (e.g. “I said I've got a lot of pain and I can't sleep with it. What do I do? ‘Learn to live with it’.”) [7]. While some studies have reviewed the literature on sleep disorders in OA, these studies, like the literature itself, were few in number and did not provide meta-analysis results on prevalence [8].

Although a significant proportion of patients report sleep disorders, this issue is rarely addressed in recommendations on OA management, and the prevalence of sleep disorders in OA patients has not been systematically assessed. It is important to know the exact frequency and types of sleep disorders affecting patients with osteoarthritis, in order to raise awareness among physicians of these symptoms as part of the overall management of the condition. Understanding the prevalence of sleep disorders in patients with osteoarthritis could lead to these disorders being considered and changes being made to patient care. We therefore conducted a systematic literature review (SLR) and meta-analysis to determine the prevalence of sleep disorders in OA patients, and their impact on OA severity according to patient-reported outcomes (PROs).

## 2 Methods

This SLR of was conducted in accordance with the PRISMA guidelines [9]. The research protocol was registered with PROSPERO (CRD120251067310).

### 2.1 Literature search

We conducted a comprehensive search across four databases: MEDLINE via NCBI PubMed, EMBASE, Web of Science and the Cochrane Library. This identified all studies related to OA and sleep disorders from inception to 22 July 2025. The search strategy comprised terms relating to two key concepts: 'osteoarthritis' and 'sleep disorders'. Keywords and Medical Subject Headings were combined for each concept. Detailed search equations are available in Supplementary File 1. Additionally, from 2020 to 2025, data were collected from the electronic abstract databases of the annual scientific meetings of the European Alliance of Associations for Rheumatology (EULAR), the American College of Rheumatology (ACR), and the Osteoarthritis Research Society International (OARSI), using the term “sleep”. The database and congress abstract searches were supplemented by a manual search, i.e. by reading the references of the included studies.

### 2.2 Eligibility criteria

The included studies were either observational cohort studies monitoring sleep disorders in OA patients, or case/control studies considering the characteristics of OA patients, whatever the location of OA with or without sleep disorders. The following sleep disorders were identified: insomnia, sleep apnea, restless legs syndrome, non-rested sleep, poor sleep quality, excessive daytime sleepiness and unspecified sleep disorders. Poor sleep quality could be defined either by the patients themselves, who reported poor sleep quality, or by a visual analog scale (VAS) ranging from 0 to 10, with 0 corresponding to the poorest sleep quality, or finally by a Pittsburgh Sleep Quality Index (PSQI) score greater than five (see below). Commentaries, protocols, editorials, case reports, or for which the full text was not available, were excluded. Although we searched for a control group to compare data between OA patients and controls, a study without a control group (a cohort study for instance) could also be included. Our search was restricted to original articles published in English or French. Review articles were excluded.

### 2.3 Study selection

The records were imported into the Rayyan software [10] and the duplicates were removed. SM initially selected potentially relevant articles based on their titles, keywords and abstracts, and then reviewed the full texts. Two additional authors (JS and ACR) oversaw the selection process, resolving discrepancies and confirming the inclusion and exclusion of studies to ensure that no relevant studies were overlooked.

### 2.4 Data extraction

One reviewer (SM) extracted all the data using a standardised data abstraction form. In cases of doubt, validation was checked by a second reader (JS).

Patients’ characteristics. Where available, we collected the following data for each article:

- the age and sex/gender of patients;
- the body mass index (BMI);
- the number of patients with depression or catastrophising, as determined by the Pain Catastrophizing Scale (PCS) [11];
- the pain scales; Pain measures could be a visual analogue scale (VAS) or a numerical rating scale (NRS) between 0 and 10, with 10 corresponding to the highest pain intensity. The NRS and VAS results were then combined to provide a single result. We also extracted the WOMAC pain score [12].
- the total WOMAC score [12].

For all extracted data, a central value (mean or median) and variability (standard deviation or interquartile range) were obtained.

Frequency of sleep disorders. n observational studies, we extracted data on the number of patients experiencing sleep disorders (e.g. insomnia, sleep apnea, restless legs syndrome, non-restorative sleep, poor sleep quality, excessive daytime sleepiness, and unspecified sleep disorders), as well as the total number of patients included in the study.

Characteristics of sleep. Where available, we extracted data on sleep quality. These may be scales such as the PSQI [min-max: 0–21] [13], the Epworth Sleepiness Scale (ESS) [min-max: 0–24] [14] or the Insomnia Severity Index (ISI) [min-max: 0–28] [15], or other parameters such as mean sleep duration, sleep efficiency (with 0 % being the lowest and 100 % the highest) and VAS sleep quality (with 10 being the highest). Higher PSQI, ESS and ISI scores indicate poorer sleep quality, higher daytime sleepiness and more severe insomnia, respectively. Furthermore, a PSQI score of more than five is often used to identify poor sleepers. We also obtained a central value (mean or median) and variability (standard deviation or interquartile range) for all extracted data.

*Quality of assessment.* The forms of Newcastle-Ottawa Scale (NOS) were used to assess the quality of the included articles [16].

*Statistical analysis.* Continuous variables were expressed as the weighted mean ± standard deviation (SD), while dichotomous variables were expressed as the number of individuals and percentages. The total number of patients in each included study and the number of patients with different sleep disorders were recorded. The prevalence of sleep disorders was then calculated by meta-analysis of proportions using the inverse of the variance method and expressed as metaproportion and 95 % confidence interval (95 % CI). For continuous parameters, the differences between patients with and without sleep disorders, or between OA patients and controls, were expressed as a standardised mean difference (SMD) using the inverse variance method: moderate (0.2–0.8) and large (>0.8) [17]. The difference of proportions between patients with and without sleep disorders, or between OA patients and controls, were expressed as an odds ratio (OR). Statistical heterogeneity between the results was assessed using the I^2^ statistic, which is the most common metric for measuring the magnitude of between-study heterogeneity and is easily interpretable. I^2^ values range from 0 % to 100 %, with values below 25 % typically considered low, between 25 % and 50 % considered modest, and above 50 % considered high [18]. This statistical method generally assumes the presence of heterogeneity when the p-value of the I^2^ test is less than 0.05. Random effects models were used in cases of heterogeneity; otherwise, a fixed effects model was used. To further explore the influence of participant characteristics on prevalence estimates, univariate and multivariate metaregression analyses were conducted. The covariates included in these analyses were mean age, mean BMI, mean VAS or NRS pain, percentage of female participants, the OA location and the number of included patients in each study. The statistical analysis was conducted using Review Manager software (version 5.4), which is produced by the Cochrane Collaboration and Stata software (v.13, StataCorp, College Station, Texas, USA).

## 3 Results

### 3.1 Eligible studies

**Fig. 1:**
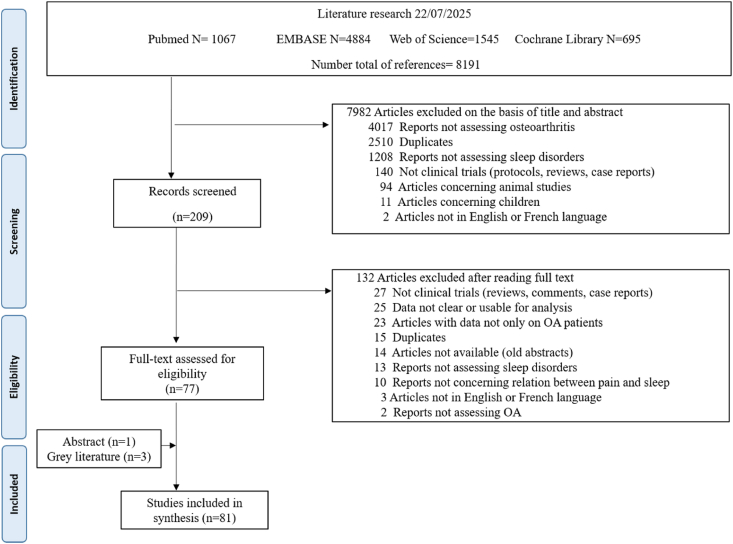
Flowchart of Manuscript selection.

Fig. 1 shows the flowchart of the publications identified in the literature search and those finally retained. After searching for and reviewing the full reports, a total of 81 eligible studies comprising 289,914 OA patients were identified (Supplementary file 2). Three additional studies were found through grey literature (Omran, Ercin and Ghauri), and one study was found through abstract archives (Mulrooney).

### 3.2 Study characteristics

The characteristics of the included studies can be found in Supplementary files 3 and their methodological quality can be found in Supplementary file 4. Most of the included studies were cross-sectional (n = 32), followed by cohort studies (n = 19) and case-control studies (n = 12). Eighteen studies were congress abstracts. A total of 75,129 patients with knee OA, 708 with hand OA and 236 with hip OA were included in the 44 37, four and two studies, respectively. Two other studies involved 621 patients with temporomandibular OA. Eight studies included knee and hip OA patients (11,039 patients), but did not provide separate information according to OA location. One study included hip and hand OA patients (Hawker 2010, 613 patients), but did not distinguish between locations. Twenty-seven studies did not specify the OA location (201,568 patients).

Sixty-three studies (276,092 patients) assessed the prevalence of sleep disorders in OA patients; twenty-three compared OA patients with controls and twelve compared patients with and without sleep disorders.

The majority of OA patients were female (66.3 % [95 % CI 64.1–68.5]; 65 studies, 282,015 patients). The weighted mean age and BMI were 65.5 ± 12.6 years (69 studies and 276,344 patients) and 28.9 ± 5.1 (38 studies and 82,474 patients), respectively. The weighted VAS pain score and WOMAC total score were 4.7 ± 2.1 (16 studies and 11,070 patients) and 37.6 ± 17.3 (6 studies and 583 patients), respectively.

### 3.3 Frequency of sleep disorders and sleep characteristics in OA patients

**Fig. 2:**
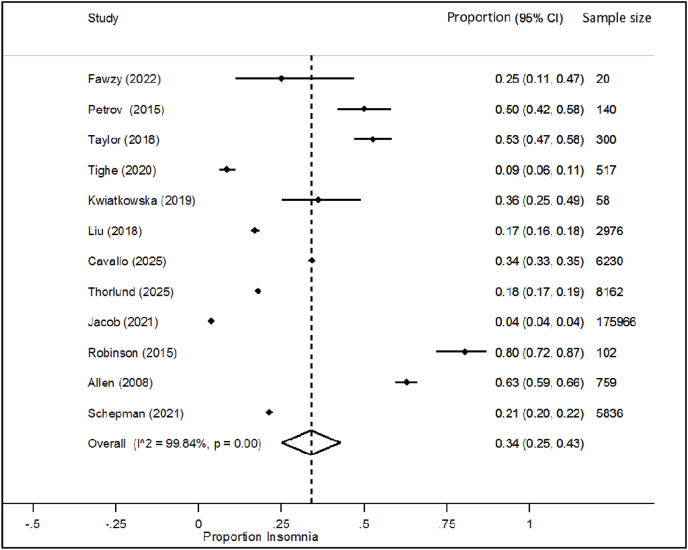
Metaproportion of insomnia in OA patients.

Of the 276,092 OA patients in the sixty-three studies, 76,553 were reported to have sleep disorders, giving a meta-proportional prevalence of sleep disorders in OA patients of 68.9 % (95%CI: 58.4 %–78.5 %) (Table 1 and supplementary file 5). Most OA patients were poor sleepers, defined as having a PSQI score higher than 5 (59.2 % (95 % CI: 44.7–73.0) across 14 studies involving 5422 patients). The weighted mean PSQI score was 8.8 ± 3.8 (19 studies; 3531 OA patients). Table 1 summarises the prevalence of different sleep disorders (e.g. sleep apnea and insomnia) and other sleep abnormalities in OA patients. Fig. 2 shows the prevalence of insomnia in OA patients.

**Table 1:** Prevalence of sleep disorders and sleep characteristics in OA patients.

	Number of studies	Number of patients	Metaproportion % [CI 95 %]	Weighted mean ± SD
Sleep disorders	63	276,092	68.9 % [58.4–78.5]	
Poor sleep (PSQI>5)	14	5422	59.2 % [44.7–73.0]	
Not rested sleep	4	5070	21.8 % [13.4–31.5]	
Daytime sleepiness	3	1440	28.8 % [11.5–50.2]	
Sleep apnea	9	238,942	32.0 % [23.2–41.4]	
Insomnia	12	201,066	34.0 % [25.0–43.0]	
Restless legs syndrome	5	3669	51.6 % [31.5–71.4]	
Less than 6 h sleep	4	4728	31.9 % [25.0–39.2]	
PSQI [min-max: 0–21]	19	3531		8.8 ± 3.8
ISI [min-max: 0–28]	4	775		10.9 ± 7.8
ESS [min-max: 0–24]	5	1315		7.3 ± 2.8
Mean hours of sleep	9	820		6.5 ± 1.9
Sleep efficiency % [min-max: 0–100]	10	892		82.5 ± 14.7
Mean sleep quality [min-max: 0–10]	4	1271		7.5 ± 2.3

SD = standard deviation; PSQI=Pittsburgh Sleep Quality Index; ESS = the Epworth Sleepiness Scale; ISI = the Insomnia Severity Index.

The prevalence of sleep disorders was found to be 70.3 % (95 % CI 67.2–73.2 %) in patients with only knee OA (29 studies; 70,027 patients), 79.5 % (95 % CI: 68.5–88.7) in patients with hand OA (four studies; 708 patients) and 73.3 % (95 % CI: 45.3–93.7) in patients with hip OA (two studies; 236 patients). In the 27 studies (involving 204,610 participants) where the OA location was not specified (18 studies) or was specified as multiple locations (nine studies involving knee and hip OA patients (eight studies) and hand and hip OA patients (one study)), the prevalence of sleep disorders was 63.9 % (95 % CI: 46.0–79.9 %). Only one study (Su et al.) examined the prevalence of sleep disorders in temporomandibular OA patients, finding it to be 74.4 % in a sample of 511 patients.

**Fig. 3:**
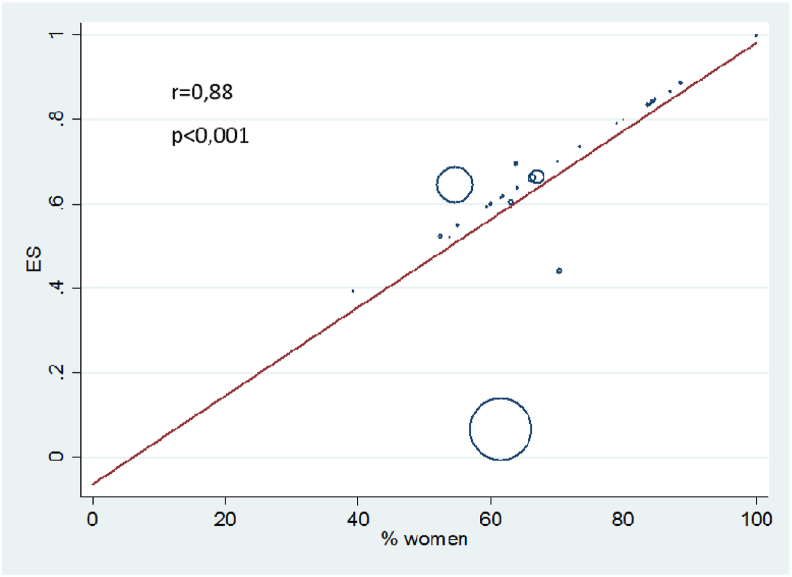
Metaregression analysis between prevalence of sleep disorders and the percentage of women r = regression coefficient; p = p-value.

In meta-regression analysis, the prevalence of sleep disorders increased with age, BMI and pain intensity, but decreased in patients with knee OA compared to other locations, though none of these changes reached statistical significance. The prevalence of sleep disorders was positively associated with the percentage of women (r = 0.88, p < 0.001) (Fig. 3) and negatively associated with the number of patients included (r = −0.30, p = 0.03). In multivariate regression analysis, the percentage of women remained positively associated with the prevalence of sleep disorders. No other significant associations were found between patients’ characteristics and insomnia, sleep apnea or restless legs syndrome.

### 3.4 Comparison of patients with and without sleep disorders

OA patients with sleep disorders were significantly younger (60.7 ± 9.0 for 1596 patients vs. 64.0 ± 8.9 for 2758 patients; p < 0.001) and had higher BMIs (31.9 ± 6.3 for 1359 patients vs. 29.5 ± 5.1 for 1714 patients; p < 0.001) than OA patients without. We found no difference in gender. The frequency of man was found to be 41.1 % (95 % CI: 23.9–60.1) in OA patients with sleep disorders (10 studies, 1596 patients) and 51.8 % (95 % CI: 32.2–71.1) in OA patients without sleep disorders (10 studies, 2758 patients) (p = 0.23).

### 3.5 Comparison of OA patients with controls

**Fig. 4:**
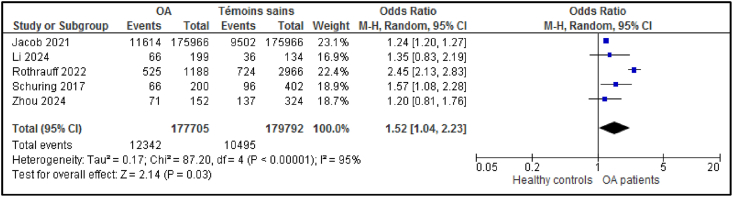
Comparison of the prevalence of sleep disorders in OA patients and healthy controls.

Only a few studies have compared the sleep characteristics of OA patients with those of healthy controls. Table 2 summarises the results of the comparison with healthy controls. OA patients had higher PSQI scores, lower sleep efficiency, and a higher prevalence of sleep disorders, sleep apnea, and being a poor sleeper (Fig. 4). Three studies compared sleep characteristics between OA and rheumatoid arthritis (RA) patients. No difference was found in PSQI (8.3 ± 4.1 for 154 OA patients vs. 7.9 ± 3.9 for 234 RA patients; p = 0.38), AIS (6.5 ± 4.6 for 78 OA patients vs. 9.0 ± 4.4 for 140 RA patients; p = 0.23) or mean hours slept (6.9 ± 1.5 for 80 OA patients vs. 5.6 ± 1.3 for 110 RA patients; p = 0.21), suggesting a similar level of sleep disturbances between OA and RA.

**Table 2:** Comparison of sleep characteristics between OA patients and healthy controls.

	Number of studies	OA group	Healthy controls group	SMD ou OR	I2	p-value
PSQI	5	n = 456	n = 549	0.78 [0.28; 1.28]	91 %	0.002
		7.9 ± 3.4	6.5 ± 2.7			
ESS	2	n = 177	n = 301	0.18 [-0.01; 0.37]	0 %	0.06
		5.4 ± 3.4	4.7 ± 3.6			
Hours of sleep	2	n = 57	n = 50	0.04 [-0.35; 0.42]	30 %	0.86
		7.0 ± 1.0	6.8 ± 1.0			
Sleep efficiency	4	n = 171	n = 141	−0.83 [-1.52; −0.14]	84 %	0.02
		82.1 ± 12.5	89.8 ± 6.0			
Sleep disorders, % [CI95 %]	5	n = 177,705	n = 179,792	OR = 1.52 [1.04; 2.23]	95 %	0.03
		31.2 % [9.6; 58.4]	23.2 % [9.5; 40.7]			
Sleep apnea, % [CI95 %]	3	n = 237,224	n = 432,311	OR = 1.84 [1.74; 1.94]	97 %	<0.001
		17.9 % [9.7; 28.0]	13.0 % [3.6; 27.1]			
Poor sleepers (PSQI>5), % [CI 95 %]	2	n = 766	n = 1947	OR = 1.98 [1.67; 2.35]	97 %	<0.001
		58.5 % [46.8; 69.7]	28.2 % [8.1; 54.5]			
Less than 6 h sleep, % [CI 95 %]	2	n = 3039	n = 11327	OR = 0.93 [0.85; 1.01]	88 %	0.09
		31.0 % [25.8; 36.4]	31.2 % [30.4; 32.1]			

OA: osteoarthritis; n = number of patients; SMD = standardized mean difference; OR = odds ratio; PSQI=Pittsburgh Sleep Quality Index; ESS = the Epworth Sleepiness Score.

### 3.6 Impact of sleep disorders in PRO

As shown in Table 3, OA patients with sleep disorders had worse all patient-reported outcomes (pain, depression and catastrophising). All these studies only involved patients with knee OA.

**Table 3:** Comparison of OA patients with and without sleep disorders.

	Number of studies	OA patients with sleep disorders	OA patients without sleep disorders	SMD (OR when specified)	p-value
Depression	3	n = 710	n = 1146	OR = 13.1 [5.73; 29.71]	<0.001
		32.5 % [26.6; 38.7]	3.8 % [2.7; 5.0]		
PCS	2	n = 217	n = 95	0.91 [0.66; 1.17]	<0.001
		18.0 ± 11.5	9.9 ± 7.4		
VAS pain [min-max: 0–10]	3	n = 331	n = 123	0.70 [0.49; 0.92]	<0.001
		4.4 ± 2.0	2.9 ± 2.0		
WOMAC pain [min-max: 0–20]	5	n = 1038	n = 2576	0.63 [0.55; 0.71]	<0.001
		5.9 ± 4.1	2.8 ± 3.1		
Total WOMAC [min-max: 0–10]	2	n = 222	n = 69	0.71 [0.43; 0.99]	<0.001
		5.4 ± 1.9	3.9 ± 1.8		

OA: osteoarthritis; n = number of patients; BMI = body mass index; SMD = standardized mean difference; OR = odds ratio; PCS= Pain catastrophizing scale; VAS = visual analog scale.

## 4 Discussion

We assessed the prevalence of sleep disorders in OA patients and found a pooled proportion of 69 %, including insomnia, sleep apnea or restless legs syndrome. The prevalence of sleep disorders was high regardless of the OA location: It was 70.3 % for knee OA, 79.5 % for hand OA, 73.3 % for hip OA, and 63.9 % for OA not specified or affecting multiple locations. The presence of sleep disorders in OA is associated with greater symptom severity or clinical impact, particularly in terms of pain. All studies assessing the impact of sleep disorders on pain or other PROs have focused on patients with knee OA. To date, no studies have examined this impact in other areas, such as the hip or hand. However, it is reasonable to suppose that the results found in patients with knee OA can be extrapolated to other OA locations. Therefore, clinicians should be aware of this high prevalence and screen for sleep disorders when managing OA patients, especially those experiencing high pain intensity. Our findings are fully consistent with the current view of OA as a serious disease that requires holistic management. This understanding takes into account not only joint symptoms, but also extra-articular symptoms and associated comorbidities.

The link between sleep disorders and OA is difficult to assess because both conditions are common in the general population. For example, 56 % of Americans and 31 % of Europeans people of the general population reported experiencing at least one symptom of insomnia several nights a week [19]. However, our comparison of OA patients and healthy controls revealed a higher prevalence of sleep disorders and sleep apnea, as well as poorer sleep quality, in OA patients, at the same extent of RA. Therefore, we can conclude that OA associated with sleep disorders. Despite the growing recognition of the link between OA and sleep problems, the underlying mechanisms are not fully understood [20]. The design, patient population, definitions of sleep disorders and outcome measures of previous studies vary widely, making it difficult to draw definitive conclusions. Recent evidence suggests that central sensitization, involving synaptic plasticity and neuronal hyperexcitability in the central nervous system, plays a key role in maintaining chronic pain in OA [21]. Sleep disorders can exacerbate this hypersensitivity, creating a two-way relationship between pain and poor sleep. [[22], [23], [24]]. Furthermore, neurogenic inflammation, which is characterized by glial cell activation and increased production of pro-inflammatory cytokines in the central nervous system, has been linked to the pathophysiology of chronic pain and sleep disorders associated with OA. This neuroinflammatory response can disrupt the neuronal circuits responsible for regulating sleep, thereby perpetuating sleep disorders in affected patients [25].

Polysomnographic recordings remain the gold standard for assessing sleep quality and detecting sleep disorders, but such evaluations are expensive and difficult to access in routine care and everyday practice. Some validated questionnaires assessing the perception of sleep quality are available and provide relevant information. The PSQI comprises 19 self-assessment questions, while the ESS consists of eight questions that evaluate sleep quality and the likelihood of dozing off in various situations, respectively. A PSQI score higher than five indicates poor sleep quality and can be used to identify individuals with sleep disorders, which can then be treated specifically. These easy-to-use screening tools could also be integrated into patient assessments. Improving awareness and providing doctors with better training on sleep disorders and how to use relevant scales, such as PSQI and ESS, could help to detect and manage sleep disorders in OA patients more effectively. Using connected devices to measure sleep parameters and detect sleep disorders could make assessing the sleep quality of patients with osteoarthritis more efficient.

We found a higher prevalence of obstructive sleep apnea in OA patients than in healthy controls. Is OA itself a risk factor for sleep apnea? This question is difficult to answer because age and obesity are common risk factors for both OA and sleep apnea, as well as being possible confounding factors [[26], [27], [28]]. However, obesity may be the most important causal factor because we found OA patients with sleep disorders to have a higher BMI and lower age than those without sleep disorders. Body mass index was found to mediate 36.9 % (95 % CI, 4.64–73.2 %, *p* = 0.026) of the obstructive sleep apnea effects on OA risk [29]. Therefore, in line with all recommendations for the management of OA [[30], [31], [32], [33]], combating obesity and implementing dietary measures and promoting regular physical activity to lose weight could also prove effective in limiting the occurrence of sleep disorders and reducing patients' pain and improving their quality of life.

We found that people with OA displaying sleep disorders experienced higher pain intensity and total WOMAC scores, as well as a higher frequency of catastrophising and depression, namely a more severe clinical presentation of OA. Different studies suggest that the relationship between pain and sleep is bidirectional in OA. Improving sleep quality could reduce pain intensity, while effective pain relief could lead to better sleep. Vitiello et al. demonstrated this association, concluding that short-term improvements in insomnia symptoms predict long-term improvements in sleep, pain and fatigue [34]. The relationship between depression, pain catastrophising, and sleep disorders in OA was recently reported by Wilk et al. [35]. Fatigue is also a common complaint among patients with osteoarthritis. Tang et al. concluded that fatigue was the strongest and most consistent predictor of night-time sleep complaints and daytime sleep-related consequences, regardless of the scale used to measure these concepts [36]. Therefore, the care of a patient with osteoarthritis must include an assessment of the following symptoms: pain, fatigue, sleep, depression and catastrophising. All of these symptoms are interrelated [[37], [38], [39], [40]]. Chronic pain can lead to physical fatigue because it puts constant stress on the body. Poor sleep and physical exertion can exacerbate pain. Poor sleep can also lead to mood disturbances, and conversely, depression can make it harder to sleep. Experiencing chronic pain can lead to feelings of frustration, helplessness and sadness, which can result in depression. Depression can make pain feel more intense or harder to manage, further reducing the patient's ability to cope. Fatigue can exacerbate negative expectations and fears about the progression of a patient's disease. When patients feel constantly drained and unable to function, they may begin to catastrophise, assuming that their condition will only worsen and that they will be unable to manage it. As illustrated above, when assessing a patient with OA, healthcare providers need to recognise that symptoms such as pain, fatigue, depression, sleep disturbances and catastrophising do not exist in isolation. Taking a multidimensional approach that addresses the physical, emotional, and psychological aspects of OA can improve overall outcomes. Effectively managing OA requires recognising how one symptom can influence another, and understanding this interrelationship is key to providing holistic care.

It is important to screen for and diagnose sleep disorders because recent international recommendations have been published to improve the management of insomnia [41], restless legs syndrome [42] and sleep apnea [43]. No et al. conducted a SLR investigating sleep treatments for patients with OA pain. They concluded that cognitive behavioural therapy and certain pharmacological treatments could alleviate both sleep disorders and pain [44]. The high prevalence of sleep disorders in OA patients, their impact on PROs, and the potential for improvement through pharmacological or non-pharmacological therapies, suggests that they should be included in the overall care of OA patients and considered as extra-articular signs of osteoarthritis. The first step in treating sleep disorders may involve non-pharmacological therapies. These include making changes to your lifestyle and diet, such as maintaining regular wake-up and bedtimes during the week and at weekends, doing at least 2.5 h of physical activity per week, moderating your consumption of stimulants, such as coffee, tea, cola and energy drinks (and avoiding them after 2 p.m.), creating a bedroom environment that is conducive to sleep in terms of darkness, silence and temperature (i.e. between 18 and 20 °C or 64 and 68 °F), limiting your alcohol consumption (especially in the evening) and establishing calming rituals, such as reading, drawing, drinking herbal tea or meditating, to help your body transition to a sleep-conducive rhythm. If these measures do not improve sleep, cognitive behavioural therapy may be considered, either on its own or in combination with pharmacological treatments such as benzodiazepines, daridorexant, and low-dose sedative antidepressants, depending on the sleep disorder reported by the patient [[41], [42], [43]].

Our study has some limitations. Firstly, quite a small number of studies assessing the prevalence of common sleep disorders such as insomnia and obstructive sleep apnea were included in this meta-analysis. This suggests that this common issue is under-researched and that awareness needs to be raised within the medical community. While this meta-analysis is insufficient to draw definitive conclusions or provide an accurate estimate of the prevalence of sleep disorders in OA, the data from around 10,000 OA patients suggests that the prevalence is significant and that OA is associated with sleep disorders. There is also some publication bias, and we cannot rule out the possibility that some studies were not published due to insufficient original results or too few participants.

Sleep disorders are common among people with OA and can significantly affect the severity of pain and functional impairment, particularly in cases of knee OA. A systematic assessment of sleep disorders in OA patients, along with targeted management, should be integrated into future therapeutic recommendations to improve overall patient care, regardless of OA location. To date, no studies have examined this impact in other joints. While it is reasonable to suppose that the results found in patients with knee OA can be extrapolated to other OA locations, this needs to be confirmed by future studies.

## Author contributions

All authors made a significant contribution to the work reported, whether that is in the conception, study design, execution, acquisition of data, analysis and interpretation, or in all these areas; took part in drafting, revising or critically reviewing the article; gave final approval of the version to be published; have agreed on the journal to which the article has been submitted; and agree to be accountable for all aspects of the work.

## Role of the funding source

This research received no funding.

## Conflict of interest

SM declares that he received personal fees from Bristol Myers Squibb, Pfizer, Abbvie, Novartis, Roche, Chugai, Merck, Sharp, and Dohme, Tilman, although unrelated to the submitted work.

J.S. declares that he received personal fees from MSD, Pfizer, Abbvie, Fresenius Kabi, Grünenthal, BMS, Lilly, Novartis, AstraZeneca, Nordic Pharma, IBSA, Guerbet, UCB and Janssen, and research grants from Pfizer.

ACR declares that she has no competing interest.
