# Supplementary material for: Prevalence and impact of sleep disorders in osteoarthritis: A systematic review and meta-analysis
Source: Osteoarthr Cartil Open. 2025 Dec 24;8(1):100738. doi: 10.1016/j.ocarto.2025.100738 (PMC12805363; doi:10.1016/j.ocarto.2025.100738)

**Supplementary files**

**Supplementary file 1. Search equations**

Medline via PubMed: ("Osteoarthritis"[mesh] OR "Osteoarthritis"[tiab] OR "Osteo-arthritis"[tiab] OR osteoarthr*[tiab] OR osteo-arthr*[tiab] OR "osteoarthrosis"[tiab] OR "osteoarthroses"[tiab] OR "degenerative arthritis"[tiab] OR "arthrosis"[tiab] OR "arthroses"[tiab]) AND (“dyssomnias”[mesh] OR “dyssomnias”[tiab] OR “sleep”[mesh] OR “sleep”[tiab] OR “sleep apnea syndromes”[mesh] OR “apnea”[tiab] OR “bruxism”[tiab] OR “bruxism”[mesh] OR “restless”[tiab] OR “sleep initiation and maintenance disorders”[mesh] OR “insomnia”[tiab]).

Embase: ('osteoarthritis'/exp OR 'osteoarthritis':ti,ab,kw OR 'osteo-arthritis':ti,ab,kw OR 'osteoarthr*':ti,ab,kw OR 'osteo-arthr*':ti,ab,kw OR 'osteoarthrosis':ti,ab,kw OR 'osteoarthroses':ti,ab,kw OR 'degenerative arthritis':ti,ab,kw OR 'arthrosis':ti,ab,kw OR 'arthroses':ti,ab,kw) AND ('dyssomnia'/exp OR 'dyssomnias':ti,ab,kw OR 'sleep'/exp OR 'sleep':ti,ab,kw OR 'sleep apnea syndromes'/exp OR 'apnea':ti,ab,kw OR 'bruxism':ti,ab,kw OR 'bruxism'/exp OR 'restless':ti,ab,kw OR 'insomnia'/exp OR 'insomnia':ti,ab,kw).

Cochrane: (Osteoarthritis OR Osteo-arthritis OR osteoarthr* OR osteo-arthr* OR osteoarthrosis OR osteoarthroses OR degenerative arthritis OR arthrosis OR arthroses) AND (sleep OR apnea OR bruxism OR restless OR dyssomnia OR insomnia)

Web of Science: (Osteoarthritis OR Osteo-arthritis OR osteoarthr* OR osteo-arthr* OR osteoarthrosis OR osteoarthroses OR degenerative arthritis OR arthrosis OR arthroses) AND (sleep OR apnea OR bruxism OR restless OR dyssomnia OR insomnia).

**Supplementary file 2. References of the 81 included studies (in alphabetical order)**

1. Akintayo RO, Yerima A, Uhunmwangho C, Olaosebikan H, Akpabio AA. Tossing and turning with degenerative arthropathy: an assessment of poor sleep quality in knee osteoarthritis. Reumatologia 2019 ;57 : 207-13.
2. Alburquerque-García A, Rodrigues-de-Souza DP, Fernández-de-las-Peñas C, Alburquerque-Sendín F. Association between muscle trigger points, ongoing pain, function, and sleep quality in elderly women with bilateral painful knee osteoarthritis. J Manipulative Physiol Ther. 2015 ;38 : 262-8.
3. Allen KD, Bosworth HB, Coffman C, Jeffreys A, Oddone EZ, Yancy Jr, et al. Patient characteristics associated with insomnia and sleep apnea in knee and hip OA. Arthritis Rheum. 2013;65:S458-S459.
4. Allen KD, Renner JB, Devellis B, Helmick CG, Jordan JM. Osteoarthritis and sleep: the Johnston County Osteoarthritis Project. J Rheumatol. 2008;35:1102-7.
5. Aslan SN, Bozgeyik-Bagdatli S, Kinikli GI. The influence of pain characteristics on insomnia, knee function, and muscle strength in patients with knee osteoarthritis. Ann Rheum Dis. 2025;84:1607-8.
6. Azizi, D, Wetzel, S, Olave, M, Gillcrist, R, Brubeck, H, White, D, et al. Z's and Knees: Associations of Participant-reported and Objective Sleep Measures with Pain Among US Veterans with Osteoarthritis of the Knee. Arthritis Rheumatol. 2024;76:1819-22.
7. Bordvik DH, Steen Pettersen P, Gløersen M, Mulrooney E, Neogi T, Kjeken I, et al. The associations between sleep problems and pain outcomes in people with hand osteoarthritis - Data from the Nor-hand study. Osteoarthr Cartil Open. 2025;7:100579.
8. Boye Larsen, D, Laursen, M, Simonsen, O, Arendt-Nielsen, L, Petersen, KKThe association between sleep quality, preoperative risk factors for chronic postoperative pain and postoperative pain intensity 12 months after knee and hip arthroplasty. British J Pain. 2021;15:486-96.
9. Brahem M, Sarraj R, Chebil A, Mlouki I, Haddada I, Maraoui M, et al. Evaluation of sleep disorders in knee osteoarthritis (Epworth and Pittsburg scales). Médecine du sommeil. 2021;18:179-85.
10. Campbell CM, Buenaver LF, Finan P, Bounds SC, Redding M, McCauley L, et al. Sleep, Pain Catastrophizing, and Central Sensitization in Knee Osteoarthritis Patients With and Without Insomnia. Arthritis Care Res. 2015;67:1387-96.
11. Cavallo M, Badley EM, Perruccio AV. Insomnia symptoms among individuals with osteoarthritis and symptoms indicative of osteoarthritis: A population-based cross-sectional study using the CLSA. Plos One. 2025:20:e0322361.
12. Chen CJ, McHugh G, Campbell M, Luker K. Subjective and Objective Sleep Quality in Individuals with Osteoarthritis in Taiwan. Musculoskelet Care 2015:13:148-59.
13. Dai Z, Neogi T, Brown C, Nevitt M, Lewis CE, Torner J, et al. Sleep Quality Is Related to Worsening Knee Pain in Those with Widespread Pain: The Multicenter Osteoarthritis Study. J Rheumatol. 2020;47:1019-25.
14. Ercin D, Ozlü A. Prevalence and severity of the restless leg syndrome in patients with hip and knee osteoarthritis. J Contemp Med. 2022;12:631-35.
15. Fawzy RM, Abdel-Monem SM, El-Brashi AWS, Mohamed AAA. comparative study between rheumatoid arthritis and osteoarthritis regarding association of insomnia with disease status. Egypt Rheumatol Rehabil. 2022;49:4.
16. Fertelli TK, Tuncay FO. Fatigue in individuals with knee osteoarthritis: Its relationship with sleep quality, pain and depression. Pak J Med Sci. 2019;35:1040-4.
17. Francis A, Erridge S, Holvey C, Coomber R, Holden W, Rucker J, et al. Assessment of Clinical Outcomes in Patients With Osteoarthritis: Analysis From the UK Medical Cannabis Registry. J Pain Pallia Care Pharmacother. 2024;38:103-16.
18. Fu K, Makovey J, Metcalf B, Bennell KL, Zhang Y, Asher R, et al. Sleep Quality and Fatigue Are Associated with Pain Exacerbations of Hip Osteoarthritis: An Internet-based Case-crossover Study. J Rheumatol. 2019;46:1524-30.
19. Ghauri MI, Iqbal AM, Riaz SU, Rasool T, Khan N, Ishaq K, et al. Restless leg syndrome in osteoarthritis: A common but overlooked combination- A Study in south Asian population. JOREP. 2025;4:1006437.
20. Gilbert AL, Lee J, Song J, Semanik PA, Ehrlich-Jones LS, Kwoh CK, et al. Relationship Between Self-Reported Restless Sleep and Objectively Measured Physical Activity in Adults With Knee Osteoarthritis. Arthritis Care Res. 2021;73:687-92.
21. Hamdi W, Souissi MA, Lassoued Ferjani H, Maatallah K, Cherif I, Kaffel D. Sleep disturbances in chronic rheumatic diseases: Is disease activity the major determinant factor? Tun Med. 2021;99:890-7.
22. Hawker GA, French MR, Waugh EJ, Gignac MA, Cheung C, Murray BJ. The multidimensionality of sleep quality and its relationship to fatigue in older adults with painful osteoarthritis. Osteoarthr Cart. 2010;18:1365-71.
23. Henrotin Y, Acker P, Zwingelstein M, Mathy C. Cross-sectional study on the impact of symptomatic osteoarthritis on sleep quality. Ann Rheum Dis. 2024;83:2200
24. Huber FA, Gonzalez C, Kusko DA, Mickle A, Sibille KT, Redden DT,  et al. Neighborhood Disadvantage and Knee Osteoarthritis Pain: Do Sleep and Catastrophizing Play a Role? Arthritis Care Res. 2025;77:95-103.
25. Jacob L, Smith L, Konrad M, Kostev K. Association between sleep disorders and osteoarthritis: A case-control study of 351,932 adults in the UK. J Sleep Res. 2021;30:e13367.
26. Jeong JN, Kim SH, Park KN. Relationship between objectively measured lifestyle factors and health factors in patients with knee osteoarthritis: The STROBE Study. Medicine. 2019;98:e16060.
27. Kakazu VA, Pinto RZ, Dokkedal-Silva V, Fernandes GL, Araujo CGA, Pires GN, et al. Are Sleep Quality, Daytime Sleepiness, and Depression Associated with Knee Pain? A Cross-Sectional Study in Older Adults. Sleep Sci. 2025;18:e91-e96.
28. Kirkham-Wilson FJ, Westbury L, Bevilacqua G, Laskou F, Fuggle NO, Dennison E. Relationship between pain, physical activity and sleep quality among individuals with radiographic knee osteoarthritis: findings from the Hertfordshire Cohort Study. Rheumatology. 2025;64:146-47.
29. Kwiatkowska B, Kłak A, Raciborski F, Maślińska M. The prevalence of depression and insomnia symptoms among patients with rheumatoid arthritis and osteoarthritis in Poland: a case control study. Psychol Health Med. 2019;24:333-43.
30. Lapane KL, Shridharmurthy D, Harkey MS, Driban JB, Dubé CE, Liu SH. The relationship between restless sleep and symptoms of the knee: data from the Osteoarthritis Initiative. Clin Rheumatol. 2021;40:2167-75.
31. Lee, J, Chang, R, Semanik, P, Jackson, R, Dunlop, D. Objectively measured aspects of sleep in knee osteoarthritis. Osteoarthr Cart. 2018;26:S215-S216.
32. Lee S, Stone KL, Engeland CG, Lane NE, Buxton OM. Arthritis, Sleep Health, and Systemic Inflammation in Older Men. Arthritis Care Res. 2020;72:965-73.
33. Leigh TJ, Bird HA, Hindmarch I, Wright V. A comparison of sleep in rheumatic and non-rheumatic patients. Clin Exp Rheumatol. 1987;5:363-5.
34. Leigh TJ, Hindmarch I, Bird HA, Wright V. Comparison of sleep in osteoarthritic patients and age and sex matched healthy controls. Ann Rheum. Dis. 1988;47:40-2.
35. Li LD, Xie H, Lu L, Prlic H, Lacaille D, Avina-Zubieta JA, et al. Sleep Quality Is Associated with Perceived Memory Performance in Females with Osteoarthritis: A Population-based Cross-sectional Study. Arthritis Rheumatol. 2024;76:323-25.
36. Liu M, McCurry SM, Belza B, Buchanan DT, Dobra A, Von Korff M, et al. Effects of Pain, Insomnia, and Depression on Psychoactive Medication Supply in Older Adults with Osteoarthritis. Med Care. 2018;56:1024-31.
37. Liu SH, Shridharmurthy D, Harkey MS, Driban JB, McAlindon TE, Dubé CE, et al. Associations of sleep duration and self-reported health status among US adults with osteoarthritis. Osteoarthr Cart. 2019;27:S265-S265.
38. Luo X, Chen M, Xu J. Exploring the role of aging in the relationship between obstructive sleep apnea syndrome and osteoarthritis: Insights from NHANES data. Front Med. 2024;11:1486807.
39. Luo ZY, Li LL, Wang D, Wang HY, Pei FX, Zhou ZK. Preoperative sleep quality affects postoperative pain and function after total joint arthroplasty: a prospective cohort study. J Orthop Surg Res. 2019;14:378.
40. Ma G, Xu B, Wang Z, Duan W, Chen X, Zhu L, et al. Non-linear association of sleep duration with osteoarthritis among U.S. middle-aged and older adults. BMC Public Health. 2024;24:3565.
41. Magni N, Collier J, McNair P, Rice DA. Neuropathic Pain in Hand Osteoarthritis: A Cross-Sectional Study. J Clin Med. 2021;10: 4439.
42. Martinez R, Reddy N, Mulligan EP, Hynan LS, Wells J. Sleep quality and nocturnal pain in patients with hip osteoarthritis. Medicine. 2019;98:e17464.
43. Mehta N, Sancheti P, Shyam A, Patil K, Gugale S, Obaid N, et al. Sleep quality improves after total knee arthroplasty: Addressing early disturbance and risk factors. J ISAKOS. 2025;10:100373.
44. Miura T, Kunugiza Y, Ogawa S, Nakamura T, Hosono N, Okada S. Obstructive sleep Apnoea in patients with knee osteoarthritis before total knee arthroplasty and its impact on post-operative recovery of blood oxygen concentrations. Arch Orthop Trauma Surg. 2025;145:187.
45. Moldofsky H, Lue FA, Saskin P. Sleep and morning pain in primary osteoarthritis. J Rheumatol. 1987;14:124-8.
46. Mulrooney E, Neogi T, Dagfinrud HS, Hammer HB, Steen Pettersen P, Kvien TK, et al. Hand osteoarthritis phenotypes and their associations with pain and change in pain. Ann Rheum Dis. 2023;82:131
47. Nuñez M, Nuñez E, Lozano L, Segur JM, Montañana J, Segura V. Sleep quality in patients with severe knee osteroarthritis. Ann Rheum Dis. 2016;75:419.
48. Nuñez M, Nuñez E, Segur JM, Lozano L, Montañana J, Segura V, et al. Sleep health and quality of life in patients with knee osteoarthritis before and after total knee replacement. Ann Rheum Dis. 2017;76:1458-58.
49. Nuñez, M, Nuñez, E, Segur, JM, Lozano, L, Montañana, J, Segura, V, et al. Influence of physical activity and sleep on functional capacity and pain in patients with knee osteoarthritis. Ann Rheum Dis. 2017;76:1041-1041.
50. Omran ME, Saleem RI, Akram G, Ahmed Majid H, Wisam Mohammed S, Abdul Kareem Hussain T, et al. Sleep disturbances in patients with knee osteoarthritis. Med J Bas Univ. 2024;42:1-9.
51. Owens MA, Mun CJ, Hamilton KR, Hughes A, Campbell CM, Edwards RR, et al. Presurgical sleep and pain behaviors predict insomnia symptoms and pain after total knee arthroplasty: a 12-month longitudinal, observational study. Pain Med. 2023;24:1224-33.
52. Ozyurek S, Kaya E, Kaplan C, Kose O, Sivrioglu AK, Atik A, et al. The relationship between alexithymia and sleep disorders in patients with knee osteoarthritis. Acta Med Mediterr. 2013;29:555-60.
53. Parmelee PA, Tighe CA, Dautovich ND. Sleep disturbance in osteoarthritis: linkages with pain, disability, and depressive symptoms. Arthritis Care Res. 2015;67:358-65.
54. Petrov ME, Goodin BR, Cruz-Almeida Y, King C, Glover TL, Bulls HW, et al. Disrupted sleep is associated with altered pain processing by sex and ethnicity in knee osteoarthritis. J Pain. 2015;16:478-90.
55. Quartana PJ, Finan PH, Page GG, Smith MT. Effects of insomnia disorder and knee osteoarthritis on resting and pain-evoked inflammatory markers. Brain Behav Immun. 2015;47:228-37.
56. Robinson, M, Bounds, S, Buenaver, L, Smith, M. Explaining group differences in clinical pain among knee osteoarthritis patients: a moderated mediation model involving pain catastrophizing and sleep disturbance. J Pain. 2015;16:S26-S26.
57. Rothrauff B, Tang Q, Wang J, He J. Osteoarthritis is positively associated with self-reported sleep trouble in older adults. Aging Clin Exp Res. 2022;34:2835-43.
58. Sasahara T, Kaneko H, Liu L, Negishi Y, Aoki T, Adili, A, et al. Sleep disturbance in elderlies is associated with knee pain with knee osteoarthritis- The Bunkyo Health study.  Osteoarthr Cart. 2023;31:S61-S61.
59. Schepman P, Thakkar S, Robinson R, Malhotra D, Emir B, Beck C. Moderate to Severe Osteoarthritis Pain and Its Impact on Patients in the United States: A National Survey. J Pain Res. 2021;14:2313-26.
60. Schuring N, Aoki H, Gray J, Kerkhoffs GMMJ, Lambert M, Gouttebarge V. Osteoarthritis is associated with symptoms of common mental disorders among former elite athletes. ESSKA 2017;25:3179-85.
61. Sezgin, M, Yesildal, E, Sevim, S, Ankarali, H, Sahin, G. Sleep quality in patients with knee osteoarthritis. Ann Rheum Dis. 2017;76:977-77.
62. Silva A, Mello MT, Serrão PR, Luz RP, Ruiz F, Bittencourt LR, et al. Influence of Obstructive Sleep Apnea in the Functional Aspects of Patients With Osteoarthritis. JCSM. 2018;14:265-70.
63. Spira AP, Runko VT, Finan PH, Kaufmann CN, Bounds SC, Liu L, et al. Circadian rest/activity rhythms in knee osteoarthritis with insomnia: a study of osteoarthritis patients and pain-free controls with insomnia or normal sleep. Chrono Int. 2015;32:242-7.
64. Su N, Liu Y, Yang X, Shen J, Wang H. Association of malocclusion, self-reported bruxism and chewing-side preference with oral health-related quality of life in patients with temporomandibular joint osteoarthritis. Int Dent J. 2018;68:97-104.
65. Taylor SS, Hughes JM, Coffman CJ, Jeffreys AS, Ulmer CS, Oddone EZ,, et al. Prevalence of and characteristics associated with insomnia and obstructive sleep apnea among veterans with knee and hip osteoarthritis. BMC Musculoskelet Disord. 2018;19:79.
66. Taylor-Gjevre RM, Gjevre JA, Nair B, Skomro R, Lim HJ. Components of sleep quality and sleep fragmentation in rheumatoid arthritis and osteoarthritis. Musculoskelet Care. 2011;9:152-9.
67. Thorlund JB, Skarpsno ES, Vestergaard JJ, Skou ST, Grønne DT, Roos EM, et al. Sleep problems and insomnia are common and associated with pain intensity, number of comorbidities and analgesic use in patients with knee and hip osteoarthritis: a cross-sectional study using data from the good life with osteoarthritis in Denmark (GLA:D(®)) registry. Rheumatol Int. 2025;45:123.
68. Tighe CA, Youk A, Ibrahim SA, Weiner DK, Vina ER, Kwoh CK, et al. Pain Catastrophizing and Arthritis Self-Efficacy as Mediators of Sleep Disturbance and Osteoarthritis Symptom Severity. Pain Med. 2020;21:501-10.
69. Vela J, Dreyer L, Petersen KK, Arendt-Nielsen L, Duch KS, Amris K, et al. Quantitative sensory testing, psychological profiles and clinical pain in patients with psoriatic arthritis and hand osteoarthritis experiencing pain of at least moderate intensity. Eur J Pain. 2024;28:310-21.
70. Venturini P, Sette FE, Da Silva MG, Casonato NA, Mattiello SM. Association between sleep quality and peripheral and central pain sensitization in people with knee osteoarthritis. Osteoarthr Cart. 2024;32:S228.
71. Wang Y, Li X, Zhang Y, Ma Y, Xu S, Shuai Z, Pan F, Cai GAssociation of Sleep Disturbance With Catastrophizing and Knee Pain: Data From the Osteoarthritis Initiative. Arthritis Care Res. 2023;75:2134-41.
72. Whibley D, Braley TJ, Kratz AL, Murphy SL. Transient Effects of Sleep on Next-Day Pain and Fatigue in Older Adults With Symptomatic Osteoarthritis. J Pain. 2019;20:1373-82.
73. Whibley, D, Kratz, AL, Murphy, SL. Sleep Efficiency Moderates the Next-day Association between Physical Activity and Pain Intensity among Adults with Knee Osteoarthritis. J Pain. 2022;23:59.
74. Wilcox S, Brenes GA, Levine D, Sevick MA, Shumaker SA, Craven T. Factors related to sleep disturbance in older adults experiencing knee pain or knee pain with radiographic evidence of knee osteoarthritis. J Am Geriatr Soc. 2000;48:1241-51.
75. Xu T, Jia X, Chen S, Xie Y, Tong KK, Iezzi T, et al. Physical activity and sleep differences between osteoarthritis, rheumatoid arthritis and non-arthritic people in China: objective versus self report comparisons. BMC Public Health. 2021;21:1821.
76. Yang Z, Lv T, Jin L, Lv X, Zhu X, Wang X, et al. The relationship between obstructive sleep apnea and osteoarthritis: evidence from an observational and Mendelian randomization study. Front Neurol. 2024;15:1425327.
77. Yap AU, Zhang XH, Cao Y, Fu KY. Degenerative temporomandibular joint diseases and their relation with sleep and emotional disturbance. Cranio. 2024;42:762-69.
78. Yeung WK, Morgan K, Mckenna F. Comparison of sleep structure and psychometric profiles in patients with fibromyalgia, osteoarthritis and healthy controls. J Sleep Res. 2018;27:290-8.
79. Zautra AJ, Fasman R, Parish BP, Davis MC. Daily fatigue in women with osteoarthritis, rheumatoid arthritis, and fibromyalgia. Pain. 2007;128:128-35.
80. Zhou X, Gong Y. Exploration in association between vitamin D, sleep quality, and osteoarthritis: A modeling study. Medicine. 2024;103:e40021.
81. Zhu Y, Jiang Q, Zhang Y, Xie D, Yang Z, Lu N, et al. Osteoarthritis and the risk of sleep apnea: a general population-based cohort study. Clin Rheumatol. 2025;44:2109-117.

**Supplementary file 3. Characteristics of the 81 included studies.**

| Name of the first author | Reference journal | N patients | Outcomes | Part 1 | Part 2 | Part 3 |
| --- | --- | --- | --- | --- | --- | --- |
| Akintayo | Reumatologica 2019 | 250 | Poor sleepers, SD | ■ | ■ |  |
| Alburquerque-Garcia | J Manipulative Physiol Ther 2015 | 18 | PSQI | ■ |  | ■ |
| Allen | J Rheumatol 2008 | 759 | DS, insomnia, not rested sleep, SD | ■ |  |  |
| Allen * | ACR congress 2013 | 300 | ISI, SD | ■ |  |  |
| Aslan * | ACR congress 2025 | 33 | ISI, SD | ■ |  |  |
| Azizi * | ACR congress 2024 | 206 | Poor sleepers | ■ | ■ |  |
| Bordvik | Osteoarthritis Cart Open 2025 | 299 | SD | ■ |  |  |
| Boye Larsen | Br J Pain 2021 | 163 | PSQI, poor sleepers, SD | ■ | ■ |  |
| Brahem | Medecine du sommeil 2021 | 66 | PSQI, ESS, DS, poor sleepers, SD | ■ |  |  |
| Campbell | Arthritis Care Res 2015 | 149 | Insomnia |  | ■ |  |
| Cavallo | Plos One 2025 | 6230 | Insomnia | ■ |  |  |
| Chen | Musculoskelet Care 2015 | 192 | PSQI, hours slept, sleep efficiency, poor sleepers, SD | ■ |  |  |
| Dai | J Rheumatol 2020 | 2329 | Not rested sleep, poor sleepers, SD | ■ | ■ |  |
| Ercin | J Contemp Med 2022 | 201 | RLS, SD | ■ |  |  |
| Fawzy | Egyptian Rheumatol Rehabil 2022 | 20 | Insomnia, SD | ■ |  | ■ |
| Fertelli | J Med Sci 2019 | 151 | PSQI, poor sleepers, SD | ■ |  | ■ |
| Francis | J Pain Palliative Care Pharm 2024 | 77 | Sleep quality, SD | ■ |  |  |
| Fu | J Rheumatol 2019 | 130 | PSQI, hours slept, L6, poor sleepers, SD | ■ |  |  |
| Ghauri | J Orthop Rep 2025 | 260 | RLS, SD | ■ |  |  |
| Gilbert | Arthritis Care Res 2021 | 1892 | Not rested sleep | ■ | ■ |  |
| Hamdi | Tunisie médicale 2021 | 40 | SD | ■ |  | ■ |
| Hawker | Ostearthritis Cart 2010 | 613 | PSQI, poor sleepers, RLS, SD | ■ |  |  |
| Henrotin * | ACR congress 2024 | 175 | SD | ■ |  |  |
| Huber | Arthritis Care Res 2025 | 140 | Sleep efficiency, SD | ■ |  |  |
| Jacob | J Sleep Res 2021 | 175966 | SD | ■ |  | ■ |
| Jeong | Medicine 2019 | 52 | Hours slept, SD | ■ |  |  |
| Kakazu | Sleep Sci 2024 | 160 | PSQI, ESS, SD | ■ |  | ■ |
| Kirkham-Wilson * | Conference 2025 | 169 | Poor sleepers, SD | ■ |  |  |
| Kwiatkowska | Psy Health Med 2019 | 58 | Insomnia, SD | ■ |  | ■ |
| Lapane | Clin Rheumatol 2021 | 2517 | RLS | ■ | ■ |  |
| Lee | Arthritis Care Res 2021 | 615 | DS, L6, poor sleepers | ■ |  | ■ |
| Lee * | OARSI congress 2018 | 184 | Hours slept, sleep efficiency, SD | ■ |  |  |
| Leigh | ARD 1988 | 14 | Sleep efficiency | ■ |  | ■ |
| Leigh | Clin Exp Rheumatol 1987 | 90 | Not rested sleep | ■ |  |  |
| Li * | ACR congress 2024 | 199 | SD | ■ |  | ■ |
| Liu | Med Care 2018 | 2976 | Insomnia, SD | ■ |  |  |
| Liu * | OARSI congress 2019 | 1559 | L6 | ■ |  |  |
| Luo | J Orthop Surg Res 2019 | 994 | PSQI, ESS, sleep quality, SA, SD | ■ |  |  |
| Luo | Front Med 2024 | 1027 | SA, SD | ■ |  | ■ |
| Ma | BMC Public Health 2024 | 2424 | L6, SD | ■ |  | ■ |
| Magni | J Clin Med 2021 | 121 | PSQI, poor sleepers, SD | ■ |  |  |
| Martinez | Medicine 2019 | 106 | PSQI, SA, SD | ■ |  |  |
| Mehta | J ISAKOS 2025 | 104 | PSQI, SD | ■ |  |  |
| Miura | Arch Orthop Traum Surg 2025 | 240 | SA, SD | ■ |  |  |
| Modolfsky | J Rheumatol 1987 | 15 |  |  |  |  |
| Mulrooney * | EULAR 2023 | 213 | SD | ■ |  |  |
| Nunez * | ACR congress 2016 | 142 | SD | ■ | ■ |  |
| Nunez * | ACR congress 2017. N°SAT0703 | 453 | SD | ■ |  |  |
| Nunez n°2 * | ACR congress 2017. N°AB1150 | 105 | SD | ■ |  |  |
| Omran | Med J Bas Univ 2024 | 102 | SD | ■ |  |  |
| Owens | Pain Med 2023 | 109 | ISI, SD | ■ |  |  |
| Ozyurek | Acta Medica Med 2013 | 305 | PSQI, poor sleepers, SD | ■ |  |  |
| Parmalee * | Arthritis Care Res 2015 | 367 | SD | ■ |  |  |
| Petrov | J Pain 2015 | 150 | Insomnia | ■ |  |  |
| Quartana | Brain Behav Immun 2015 | 80 | Insomnia |  | ■ | ■ |
| Robinson * | J Pain 2015 | 102 | Insomnia, SD | ■ |  |  |
| Rothrauff | Aging Clin Exp Res 2022 | 1188 | SD | ■ |  |  |
| Sasahara * | OARSI congress 2023 | 1145 | SD | ■ |  |  |
| Schepman | J Pain Res 2021 | 5836 | Insomnia, SD | ■ |  |  |
| Schuring | Knee Surg Sports Trauma Arthrosc 2017 | 200 | SD | ■ |  | ■ |
| Sezgin * | ACR congress 2017 | 100 | Sleep efficiency | ■ |  | ■ |
| Silva | J Clin Sleep Med 2018 | 30 | Hours slept, sleep efficiency | ■ | ■ |  |
| Spira | Chronobiol Int 2015 | 142 | SD | ■ | ■ |  |
| Su | Int Dent J 2018 | 511 | SD | ■ |  |  |
| Taylor | BMC Musculosk Dis 2018 | 300 | ISI, SA, insomnia, SD | ■ | ■ |  |
| Taylor-Gjevre | Musculoskelet Care 2011 | 78 | PSQI, ESS, SA, RLS, SD | ■ |  | ■ |
| Thorlund | Rheumatol Int 2025 | 8162 | Insomnia, SD | ■ |  |  |
| Tighe | Pain Med 2020 | 517 | Insomnia, SD | ■ |  |  |
| Vela | Eur J Pain 2024 | 75 | PSQI, SD | ■ |  |  |
| Venturini * | OARSI congress 2024 | 52 | PSQI, SD | ■ |  |  |
| Wang | Arthritis Care Res 2023 | 3813 | SD | ■ |  |  |
| Whibley | J Pain 2019 | 160 | Hours slept, sleep efficiency, sleep quality, SD | ■ |  |  |
| Whibley * | J Pain 2022 | 116 | Poor sleepers, SD | ■ |  |  |
| Wilcox | JAGS 2000 | 429 | SD | ■ |  |  |
| Xu | BMC Public Health 2021 | 40 | Hours slept, sleep efficiency, sleep quality | ■ |  | ■ |
| Yang | Front Neuro 2024 | 1557 | SA, SD | ■ |  | ■ |
| Yap | Cranio 2024 | 110 | PSQI |  |  | ■ |
| Yeung | J Sleep Res 2018 | 17 | PSQI, ESS, hours slept, sleep efficiency | ■ |  | ■ |
| Zautra | Pain Med 2007 | 76 | PSQI, SD | ■ |  | ■ |
| Zhou | Medicine 2024 | 152 | SD | ■ |  | ■ |
| Zhu | Clin Rheumatol 2025 | 58674 | SA, SD | ■ |  |  |

*= abstract. Part 1: prevalence in OA; part 2: comparison of OA patients with or without sleep disorders; part 3: comparison of OA patients with controls. OA: osteoarthritis; PSQI= Pittsburgh Sleep Quality Index; ESS=the Epworth Sleepiness Score; ISI= Insomnia Severity Index; SA= sleep apnea; DS= daytime sleepiness; L6= less than 6 hours slept; RLS= rest less syndrome; SD= sleep disorders.

Supplementary file 4. Assessment of the methodological quality of the included studies. (Abstract was not assessed)

| Authors |  | Q1 | Q2 | Q3 | Q4 | Q5 | Q6 | Q7 | Q8 | Q9 |
| --- | --- | --- | --- | --- | --- | --- | --- | --- | --- | --- |
| Akintayo 2019 | Cs |  |  |  |  |  |  |  |  |  |
| Alburquerque-Garcia 2015 | C |  |  |  |  |  |  |  |  |  |
| Allen 2008 | Cs |  |  |  |  |  |  |  |  |  |
| Allen 2013 | Abstract | | | | | | | | | |
| Aslan 2025 | Abstract | | | | | | | | | |
| Azizi 2024 | Abstract | | | | | | | | | |
| Bordvik 2025 | Cs |  |  |  |  |  |  |  |  |  |
| Boye Larsen 2021 | Cs |  |  |  |  |  |  |  |  |  |
| Brahem 2021 | Cs |  |  |  |  |  |  |  |  |  |
| Campbell 2015 | C |  |  |  |  |  |  |  |  |  |
| Cavallo 2025 | Co |  |  |  |  |  |  |  |  |  |
| Chen 2015 | Cs |  |  |  |  |  |  |  |  |  |
| Dai 2020 | Co |  |  |  |  |  |  |  |  |  |
| Ercin 2022 | Cs |  |  |  |  |  |  |  |  |  |
| Fawzy 2022 | C |  |  |  |  |  |  |  |  |  |
| Fertelli 2019 | Cs |  |  |  |  |  |  |  |  |  |
| Francis 2024 | Co |  |  |  |  |  |  |  |  |  |
| Fu 2019 | Cs |  |  |  |  |  |  |  |  |  |
| Ghauri 2025 | Cs |  |  |  |  |  |  |  |  |  |
| Gilbert 2021 | Co |  |  |  |  |  |  |  |  |  |
| Hamdi 2021 | C |  |  |  |  |  |  |  |  |  |
| Hawker 2010 | Co |  |  |  |  |  |  |  |  |  |
| Henrotin 2024 | Abstract | | | | | | | | | |
| Huber 2025 | Cs |  |  |  |  |  |  |  |  |  |
| Jacob 2021 | Cs |  |  |  |  |  |  |  |  |  |
| Jeong 2019 | Cs |  |  |  |  |  |  |  |  |  |
| Kakazu 2024 | Cs |  |  |  |  |  |  |  |  |  |
| Kirkham-Wilson 2025 | Abstract | | | | | | | | | |
| Kwiatkowska 2019 | C |  |  |  |  |  |  |  |  |  |
| Lapane 2021 | Co |  |  |  |  |  |  |  |  |  |
| Lee 2021 | Cs |  |  |  |  |  |  |  |  |  |
| Lee 2018 | Abstract | | | | | | | | | |
| Leigh 1988 | C |  |  |  |  |  |  |  |  |  |
| Leigh 1987 | C |  |  |  |  |  |  |  |  |  |
| Li 2024 | Abstract | | | | | | | | | |
| Liu 2018 | Co |  |  |  |  |  |  |  |  |  |
| Liu 2019 | Abstract | | | | | | | | | |
| Luo 2019 | Co |  |  |  |  |  |  |  |  |  |
| Luo 2024 | Co |  |  |  |  |  |  |  |  |  |
| Ma 2024 | Co |  |  |  |  |  |  |  |  |  |
| Magni 2021 | Cs |  |  |  |  |  |  |  |  |  |
| Martinez 2019 | Cs |  |  |  |  |  |  |  |  |  |
| Mehta 2025 | Co |  |  |  |  |  |  |  |  |  |
| Miura 2025 | C |  |  |  |  |  |  |  |  |  |
| Modolfsky 1987 | C |  |  |  |  |  |  |  |  |  |
| Mulrooney 2023 | Abstract | | | | | | | | | |
| Nunez 2016 | Abstract | | | | | | | | | |
| Nunez 2017 | Abstract | | | | | | | | | |
| Nunez n°2 2017 | Abstract | | | | | | | | | |
| Omran 2024 | Cs |  |  |  |  |  |  |  |  |  |
| Owens 2023 | Co |  |  |  |  |  |  |  |  |  |
| Ozyurek 2013 | Cs |  |  |  |  |  |  |  |  |  |
| Parmalee 2015 | Abstract | | | | | | | | | |
| Petrov 2015 | Cs |  |  |  |  |  |  |  |  |  |
| Quartana 2015 | Cs |  |  |  |  |  |  |  |  |  |
| Robinson 2015 | Abstract | | | | | | | | | |
| Rothrauff 2022 | Co |  |  |  |  |  |  |  |  |  |
| Sasahara 2023 | Abstract | | | | | | | | | |
| Schepman 2021 | Cs |  |  |  |  |  |  |  |  |  |
| Schuring 2017 | Cs |  |  |  |  |  |  |  |  |  |
| Sezgin 2017 | Abstract | | | | | | | | | |
| Silva 2018 | Cs |  |  |  |  |  |  |  |  |  |
| Spira 2015 | Cs |  |  |  |  |  |  |  |  |  |
| Su 2018 | Cs |  |  |  |  |  |  |  |  |  |
| Taylor 2018 | Cs |  |  |  |  |  |  |  |  |  |
| Taylor-Gjevre 2011 | Cs |  |  |  |  |  |  |  |  |  |
| Thorlund 2025 | Cs |  |  |  |  |  |  |  |  |  |
| Tighe 2020 | Cs |  |  |  |  |  |  |  |  |  |
| Vela 2024 | C |  |  |  |  |  |  |  |  |  |
| Venturini 2024 | Abstract | | | | | | | | | |
| Wang 2023 | Co |  |  |  |  |  |  |  |  |  |
| Whibley 2019 | Co |  |  |  |  |  |  |  |  |  |
| Whibley 2022 | Abstract | | | | | | | | | |
| Wilcox 2000 | Co |  |  |  |  |  |  |  |  |  |
| Xu 2021 | C |  |  |  |  |  |  |  |  |  |
| Yang 2024 | Co |  |  |  |  |  |  |  |  |  |
| Yap 2024 | Cs |  |  |  |  |  |  |  |  |  |
| Yeung 2018 | C |  |  |  |  |  |  |  |  |  |
| Zautra 2007 | Cs |  |  |  |  |  |  |  |  |  |
| Zhou 2024 | Co |  |  |  |  |  |  |  |  |  |
| Zhu 2025 | Co |  |  |  |  |  |  |  |  |  |

| **Case control studies (C)** | **Cohorts studies (Co)** | **Cross-sectional studies (CS)** |
| --- | --- | --- |
| **Q1.** Is the case definition adequate ? | **Q1.** Representativeness of the exposed | **Q1.** Is the representativeness of the cases adequate? |
| **Q2.** Is the representativeness of the cases adequate? | **Q2.** Selection of the non exposed | **Q2.** Is the sample size justified and satisfactory? |
| **Q3.** Selection of controls | **Q3.** Ascertainment of exposed | **Q3.** Is the non-response rate satisfactory? |
| **Q4.** Definition of controls | **Q4.** Outcome of interest was no present at start | **Q4.** Is the ascertainment of the screening/surveillance tool validated ? |
| **Q5.** Study controls for the most important factor | **Q5.** Study controls for the most important factor | **Q5.** Are the potential confounders investigated by subgroup analysis or multivariable analysis ? |
| **Q6.** Study controls for any important factor | **Q6.** Study controls for any important factor | **Q6.** Is assessment of the outcome adequate ? |
| **Q7.** Ascertainment of outcome | **Q7.** Assessment of outcome | **Q7.** Is the statistical test clearly described and appropriate ? |
| **Q8.** Same ascertainment method for cases  and controls | **Q8.** Follow-up long enough |  |
| **Q9.** Non response rate | **Q9.** Adequacy of follow up |  |

C = case control study; CS = cross-sectional study; Co = cohort study. = Yes = No = Can’t tell = Not concerned

Supplementary file 5. Meta-proportion of sleep disorders in osteoarthritis patients.


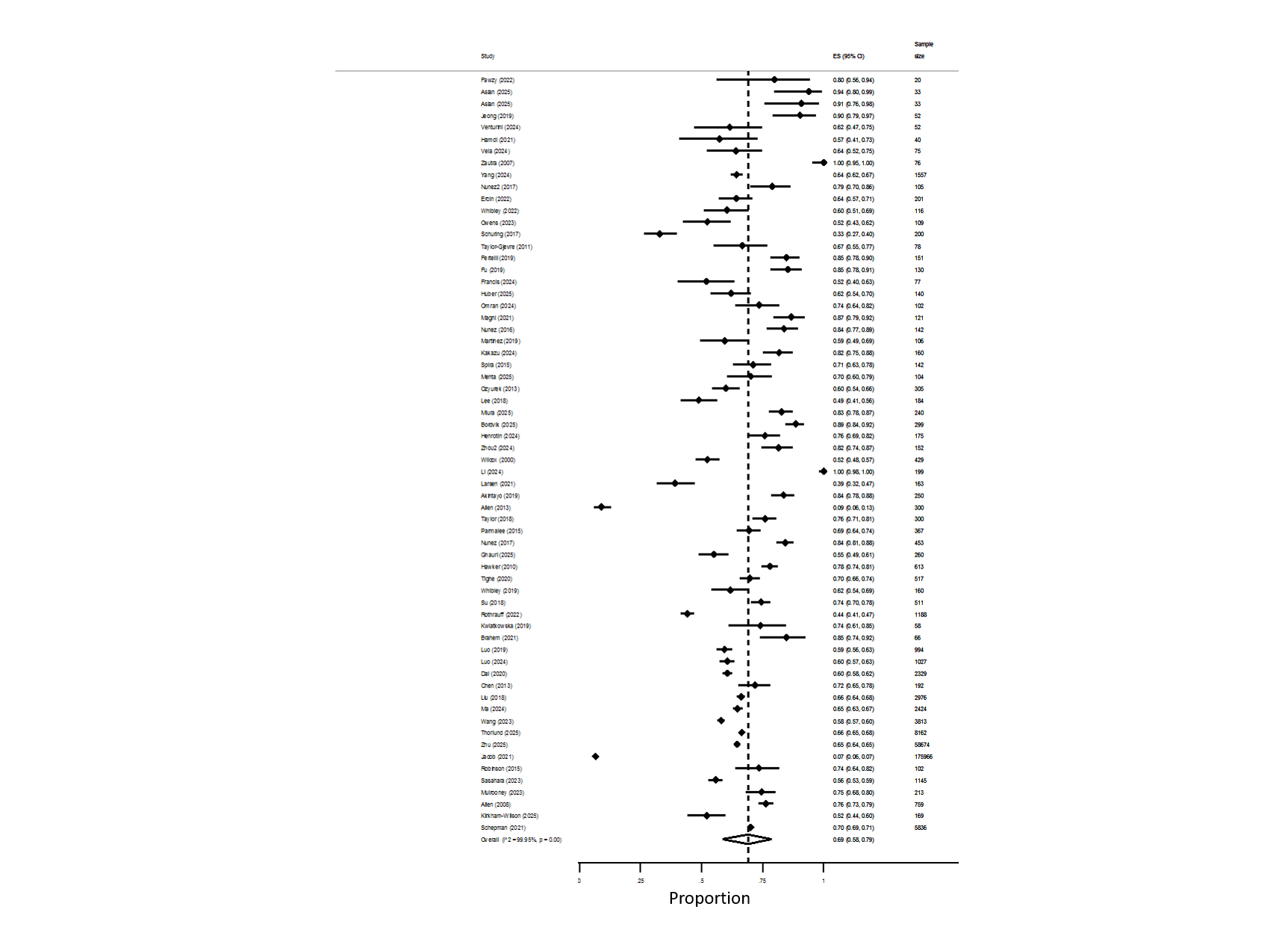

Supplement: Multimedia component 1 [file mmc1.docx]
